# Lytic activity of the virion-associated peptidoglycan hydrolase HydH5 of *Staphylococcus aureus *bacteriophage vB_SauS-phiIPLA88

**DOI:** 10.1186/1471-2180-11-138

**Published:** 2011-06-17

**Authors:** Lorena Rodríguez, Beatriz Martínez, Yuan Zhou, Ana Rodríguez, David M Donovan, Pilar García

**Affiliations:** 1 Instituto de Productos Lácteos de Asturias (IPLA-CSIC). Apdo. 85. 33300- Villaviciosa, Asturias, Spain; 2 State Key Laboratory of Agrobiotechnology, College of Biological Sciences, China Agricultural University, Beijing 100193, China; 3 Animal Biosciences and Biotechnology Lab, ANRI, ARS, USDA, 10300 Baltimore Ave, Beltsville, MD 20705-2350

## Abstract

**Background:**

*Staphylococcus aureus *is a food-borne pathogen and the most common cause of infections in hospitalized patients. The increase in the resistance of this pathogen to antibacterials has made necessary the development of new anti-staphylococcal agents. In this context, bacteriophage lytic enzymes such as endolysins and structural peptidoglycan (PG) hydrolases have received considerable attention as possible antimicrobials against gram-positive bacteria.

**Results:**

*S. aureus *bacteriophage vB_SauS-phiIPLA88 (phiIPLA88) contains a virion-associated muralytic enzyme (HydH5) encoded by *orf58*, which is located in the morphogenetic module. Comparative bioinformatic analysis revealed that HydH5 significantly resembled other peptidoglycan hydrolases encoded by staphylococcal phages. The protein consists of 634 amino acid residues. Two putative lytic domains were identified: an N-terminal CHAP (cysteine, histidine-dependent amidohydrolase/peptidase) domain (135 amino acid residues), and a C-terminal LYZ2 (lysozyme subfamily 2) domain (147 amino acid residues). These domains were also found when a predicted three-dimensional structure of HydH5 was made which provided the basis for deletion analysis. The complete HydH5 protein and truncated proteins containing only each catalytic domain were overproduced in *E. coli *and purified from inclusion bodies by subsequent refolding. Truncated and full-length HydH5 proteins were all able to bind and lyse *S. aureus *Sa9 cells as shown by binding assays, zymogram analyses and CFU reduction analysis. HydH5 demonstrated high antibiotic activity against early exponential cells, at 45°C and in the absence of divalent cations (Ca^2+^, Mg^2+^, Mn^2+^). Thermostability assays showed that HydH5 retained 72% of its activity after 5 min at 100°C.

**Conclusions:**

The virion-associated PG hydrolase HydH5 has lytic activity against *S. aureus*, which makes it attractive as antimicrobial for food biopreservation and anti-staphylococcal therapy.

## Background

Despite their relatively small size and apparent simplicity, double-stranded DNA bacteriophages propagate by a tightly programmed infection process which involves a number of steps. Adsorption of the phage to the bacterial cell wall precedes injection of the nucleic acid and subsequent DNA replication, eventually giving raise to new phage particles that are released after lysis of the host. Muralytic enzymes play essential roles in the life cycle of phages by degrading the peptidoglycan (PG) of the bacterial cell wall, facilitating the entry and eventual release of mature phage particles. Many DNA-tailed phages employ the holin-endolysin lysis system to release their progeny. Holins usually form large pores in the cytoplasmic membrane of the host allowing the endolysin to gain access to and hydrolyze the PG layer [1]. In addition to endolysins which are synthesized at the late stage of the lytic cycle, virions often harbour murein hydrolases that locally degrade the PG in order to facilitate the entry of phage DNA during infection. These virion proteins are responsible of the "lysis from without" phenomenon caused by some phages when adsorbed onto the host cell in very high numbers [2].

Virion-associated murein hydrolases appear to be widespread in bacteriophages infecting both Gram-negative and Gram-positive bacteria as shown by zymograms of fully assembled virions and homology analysis of sequenced phage/prophage genomes [3]. Several phages infecting Gram negative hosts contain hydrolytic activities at a variety of locations within the virions. A protein with N-acetylmuramidase activity is often anchored to the base plate structure, as in the T4 virion tail [4]. Similarly, a lytic endopeptidase was found to be associated with the nucleocapsid of the double-stranded RNA bacteriophage Φ6 infecting *Pseudomonas syringae *[5]. In the T7 bacteriophage, gp16 is an internal head protein with transglycosylase activity that is ejected into the cell at the initiation of infection but is required only when the cell wall is highly cross-linked [6].

The presence of muralytic activities in virions infecting Gram-positive bacteria has also been demonstrated. PG hydrolase activities have been described in the virions for *S. aureus *phages Φ11 and Φ85 [3], phiMR11 [7], P68 [8] and in the *Lactococcus lactis *phage Tuc2009 [9].

*S. aureus *is an important human pathogen that has demonstrated a unique ability to acquire antibiotic resistance traits at high frequency and can cause numerous serious diseases [http://www.medicinenet.com/staph_infection/article.htm] including food poisoning [10,11]. In the last few years, there has been a dramatic increase in the incidence of community-associated methicillin- and multi-drug-resistant *S. aureus *infections that can limit therapeutic options [12]. Therefore, there is a growing demand of new anti-staphylococcal agents.

In this context, attention has been paid to bacteriophage lytic enzymes such as endolysins and structural PG hydrolases. Purified phage endolysins have been used as therapeutics (so-called enzybiotics) against Streptococci in mice [13,14] and have been proven effective against other Gram-positive pathogens including *Enterococcus faecalis *and *E. faecium *[15], *Clostridium perfringens *[16], group B Streptococci [17], *Bacillus anthracis *[18] and *S. aureus *[19-21].

Previously, we reported the isolation of the *S. aureus *bacteriophage vB_SauS-phiIPLA88 (in short, phiIPLA88) belonging to the *Siphoviridae *family [22]. The complete genome sequence was determined (Accession number NC_011614) and zymogram analysis revealed the presence of a phiIPLA88 virion-associated muralytic enzyme [23]. In this study, we describe the structural component of phiIPLA88 particle, HydH5, which exhibits lytic activity against *S. aureus *cells. HydH5 contains a CHAP [24,25] and a LYZ2 [7] domain and the contribution of each to cell lysis has been analysed. Finally, we have determined the optimal activity conditions and heat-labile stability in order to assess HydH5's potential as an anti-*Staphylococcus *agent.

## Results

### S. aureus bacteriophage phiIPLA88 contains a structural component with a putative cell wall- degrading activity

**Figure 1:**
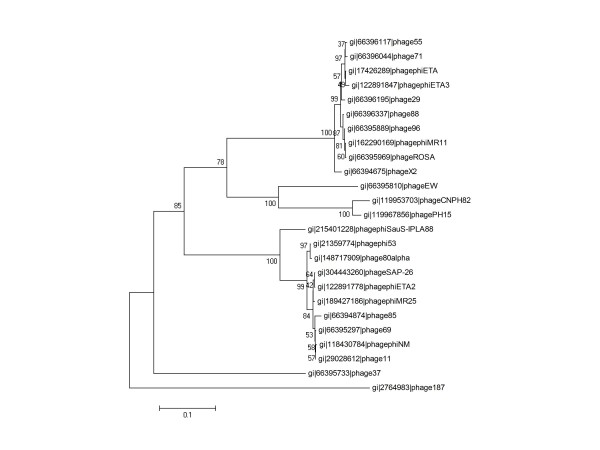
**Phylogenetic analysis of the phage phiIPLA88 virion-associated peptidoglycan hydrolase HydH5 compared to several phage peptidoglycan hydrolases**. The phylogenetic tree was constructed using the Neighbor-Joining method with 1000 bootstrap replicates and drawn to scale. The evolutionary distances were computed using the Poisson correction method and are expressed in the units of the number of amino acid substitutions per site. All positions containing gaps and missing data were eliminated from the dataset. Phylogenetic analyses were conducted in MEGA4 [53].

**Figure 2:**
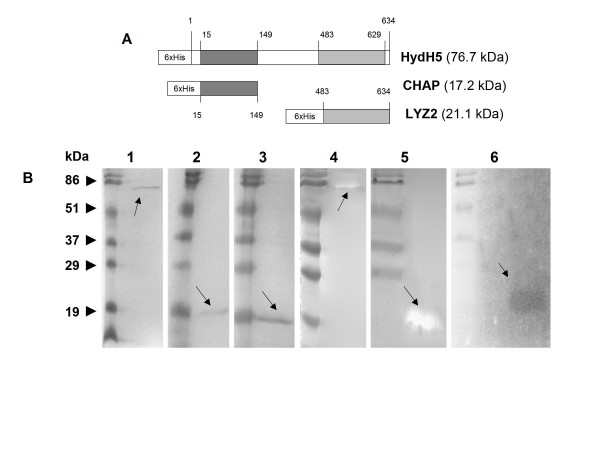
**Sequence analysis, SDS-PAGE and zymogram of the 6 × His tagged full-length HydH5 and deletion constructs**. A) Pfam domain organization of HydH5 and its deletion constructs containing CHAP (cysteine, histidine-dependent amidohydrolases/peptidases) and LYZ2 (lysozyme subfamily 2) domains. Numbers indicate the amino acid positions in HydH5. B) Comassie-blue stained SDS-PAGE gel of lane 1: purified HydH5 (76.7 kDa), lane 2: purified CHAP domain (17.2 kDa), lane 3: purified LYZ2 domain (21.1 kDa); and zymogram analysis of lane 4: purified HydH5, lane 5: crude cell extracts of induced *E. coli *clones containing CHAP domain, lane 6: crude cell extracts of induced *E. coli *clones containing LYZ2 domain. Zymograms were run with *S. aureus *Sa9 cells embedded in the gel. Molecular mass standards (Prestained SDS-PAGE Standards, broad range, BioRad Laboratories) are indicated on the left.

The virions of phage phiIPLA88 possess a structural component with lytic activity as was previously shown by zymogram analysis [23]. This lytic activity corresponded in size to that expected for the protein product of *orf58 *(72.5 kDa), which is located in the morphogenetic module with most of the phage head and tail structural genes. Computer-based similarity searches revealed that protein gp58, designated here as HydH5 (634 amino acids, Acc. Number ACJ64586), showed 91% similarity with putative PG hydrolases identified in *S*. *aureus *phi11, phiNM and phiMR25 phages (Acc. Number NP_803302.1, YP_874009.1, YP_001949862.1). A 60% similarity was detected between HydH5 and the recently characterized PG hydrolase gp61 of *S. aureus *phiMR11 phage [7]. A phylogeny tree was generated from alignment of the known staphylococcal PG hydrolases (Figure 1). The 25 different proteins were clustered into two major groups. No relation between these groups and the previous *S. aureus *phages classification based on their genome organization was observed [26]. Interestingly, PG hydrolases from phages infecting *S. epidermidis *strains (phage CNPH82 and phage PH15) were found to be very similar to those from *S. aureus *phages. Furthermore, conserved-domain analyses of HydH5 identified two typical catalytic domains found in cell wall hydrolases. At its N-terminal region (15 to 149 amino acids) a CHAP (cysteine, histidine-dependent amidohydrolase/peptidase) domain was detected [24,25]. The C-terminal region (483 to 629 amino acids) showed a LYZ2 (lysozyme subfamily 2 or glucosaminidase) [7] conserved domain. However, additional biochemical analyses of staphylococcal PG treated with HydH5 should be performed to determine the cleavage sites of each catalytic domain. The middle region of HydH5 (150 to 482 amino acids) did not show homology to any conserved sequences. Domain database and comparative sequence analysis failed to detect any known cell wall binding domain (CBD) in HydH5. A schematic of the HydH5 protein is depicted graphically later in conjunction with deletion constructs (Figure 2A).

### Predicted 3D structure of HydH5

**Figure 3:**
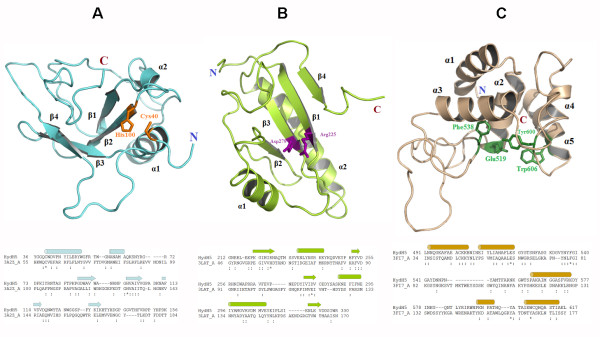
**3D structure prediction of HydH5**. Top of panels A, B and C are the predicted 3D structure of the corresponding three HydH5 domains. The structure models were generated by the MODELLER program and the cartoon representation of the structure models was prepared using Pymol (http://www.pymol.org/). Secondary structure elements and conserved catalytic residues are labelled. Bottom panels A, B and C plot the sequence alignments between three HydH5 domains and their corresponding templates. The template identification and sequence alignments were generated by the HHpred server. The probabilities of remote homologous relationship for each alignment provided by HHpred are 0.996, 0.993 and 0.996, although the sequence identities of the three alignments are only 17%, 14% and 22% respectively. Conserved residues between the three HydH5 domains and their templates are labeled by colons under the alignment if they share similar side chains, and with asterisks if identical residues. Position of α-helix and β-sheet in each domain of Hyd5 is indicated by cylinder and arrow, respectively.

The HHpred server and MODELLER program were jointly used to predict the structure of the HydH5 protein and three different domains were deduced. The predicted structure revealed similarity with the crystal structure of the *E. coli *Gsp amidase [27] belonging to the CHAP superfamily [24,25] in the N-terminal region (domain A, 36-156 amino acids), with the *Staphylococcus epidermidis *PG hydrolase AmiE [28] in the middle region (domain B, 212-326 amino acids) and with the *Listeria monocytogenes *PG hydrolase [29] in the C-terminal region (domain C, 491-617 amino acids) (Figure 3). Domain A (Gsp amidase-like domain) is predicted to have two α helices and four twisted anti- parallel β-sheets. Two conserved catalytic residues are positioned in the first α helix termini and its neighboring β-sheet (Figure 3A). A topology similar to these residues can be found in other members of this family of enzymes [27]. Domain B (N-acetylmuramoyl-L-alanine amidase-like domain) is comprised of two α helices and 4 parallel β-sheets between the helices. The topology of HydH5 catalytic residue pair is similar to the template structure but lacks any of the residues comprising the zinc binding site (Figure 3B). Domain C (mannosyl-glycoprotein endo-β-N-acetylglucosaminidase-like domain) has five predicted α helices. The conserved catalytic residue glutamine 519 was settled into the second α helix surrounded by three conserved aromatic residues, forming one side of the catalytic core (Figure 3C). However, the other side of the catalytic core usually surrounding another acidic residue is not conserved. This acidic residue is usually positioned in a β-hairpin in the template structure [29], while the structure of the corresponding region in HydH5 is predicted as a long coil rather than sheets. It is thus difficult to confidently predict where the non-conserved catalytic acidic residue settles into the predicted domain structure.

### Antimicrobial activity of PG hydrolase HydH5 and its catalytic domains

To confirm the predicted lytic activity encoded by *orf58*, the complete gene and the regions encoding the two identified catalytic domains were amplified by PCR and individually cloned into the expression vector pET-Duet1. Due to the high frequency of *E. coli *low usage codons in *orf58 *(9.15% of the total codons), HydH5 overproduction was performed in *E. coli *Rosetta (DE3) pET-Duet1-*orf58*, which carries the plasmid pRARE containing tRNA genes for six rare codons in *E. coli*. Truncated versions of HydH5 containing each of the individual catalytic domains CHAP and LYZ2 were overproduced in *E. coli *BL21(DE3)/pLysS (Figure 2B, lanes 1 to 3). Attempts to purify the HydH5 and derivative proteins after induction of *E. coli *cultures gave low yields, presumably due to their low solubility. Therefore, we proceeded to explore their recovery from inclusion bodies which were denatured and independently refolded in several buffers (see Material and Methods section). The purity of the recombinant proteins in refolding buffer was at least 95% as assessed by SDS-PAGE analysis. The proteins migrate according to their calculated molecular masses plus the 6 × His tag (76.7 kDa, 17.2 kDa, and 21.1 kDa, for the full-length HydH5, the CHAP and the LYZ2 domains, respectively) (Figure 2A).

The PG hydrolytic ability of the different lysates and purified proteins were qualitatively assayed by zymogram analysis against *S*. *aureus *Sa9 cells (Figure 2B, lanes 4 to 6). Both cell lysates and purified HydH5 showed lytic activity. However, lytic activity was only observed in the cell lysates of the catalytic domains, probably due to either a lower specific activity or a lower protein concentration of the purified truncated proteins. These results support the functionality of the putative PG hydrolytic domains found by the bioinformatic analysis. Nevertheless, their activity seems to be somewhat weaker than that shown by other staphylococcal endolysins, e.g. LysK [19,30,31], phi11 [32,33], phiMR11 [34] because when classical turbidity reduction assays were performed, neither HydH5 nor its CHAP and LYZ2 truncated derivatives were found to be active against *S*. *aureus *Sa9 cells (data not shown).

The antimicrobial activity of purified HydH5, CHAP and LYZ2 derivatives was quantified by the CFU reduction analysis. 250 μl of exponentially growing *S. aureus *Sa9 cultures (4 × 10^6 ^CFU/ml) were challenged to 20 μg of either the full-length or each truncated proteins (0.08 μg/μl, final concentration). Staphylococcal viability counts were reduced by 40.4 ± 1.5%, 25.7 ± 4.9%, and 23.1 ± 6.6%, respectively, compared with the untreated controls. Therefore, despite the fact that lysis was not detected in the zymograms with the truncated purified proteins both seemed to be active against *S. aureus *Sa9 cells.

Moreover, the susceptibility of *S. aureus *Sa9 cells to HydH5 seems to be dependent on the growth stage. Cells collected during the early and mid-exponential stages of growth were the most susceptible to the PG hydrolase HydH5 (data not shown). By contrast, challenges using late exponential and stationary growth stages cells showed a reduction around 50% in HydH5 activity (data not shown).

### HydH5 catalytic domains have cell binding capacity themselves

**Figure 4:**
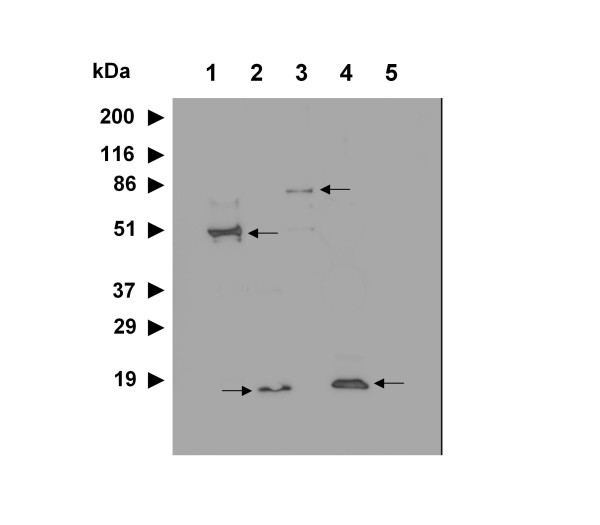
**Western blot analysis of 6 × His tagged full-length HydH5 and truncations bound to intact *S. aureus *Sa9 cells**. Purified proteins (5 μg) were mixed with exponentially growing cells, centrifuged and the pellet was washed with PBS, boiled with the sample buffer and electrophoresed in a 15% SDS-PAGE gel. Western blot analysis with monoclonal antibodies recognizing His-tags were used for detecting the cell bound proteins. Lane 1, endolysin LysH5 (53.7 kDa); lane 2, CHAP (17.2 kDa); Lane 3, HydH5 (76.7 kDa); Lane 4, LYZ2 (21.1 kDa); Lane 5, control (washed cells without protein addition).

The relative low lytic activity of the hydrolase HydH5 *in vitro *and the lack of a predicted CBD domain might suggest a poor capacity to bind to the cell wall. To assess the ability of full-length HydH5 and its truncated versions to target PG, 5 μg of each protein were added to exponentially growing *S. aureus *Sa9 cells. As a positive control, 5 μg of the phiIPLA88 endolysin LysH5 [35] was included. This protein harbours a SH3b CBD domain and specifically recognizes staphylococcal cells [35]. SDS-PAGE plus Western blot analyses showed that all the 6× His tagged protein tested (full-length HydH5 and its catalytic CHAP and LYZ2 domains, and LysH5) were able to bind staphylococcal cells, since a interaction of these proteins with His-tag specific antibodies were detected when the washed cell pellets were loaded in the gels (Figure 4). The size marker confirms the expected size of the 6× His tagged proteins previously deduced from the sequence data and, thus, the observed shadow bands could be due to unspecific antibody binding (Figure 4). As HydH5 and its truncated derivatives bind cells under these experimental conditions, a CBD domain seems not be required for PG targeting.

### HydH5 activity is inhibited by cations and is highly thermostable

**Figure 5:**
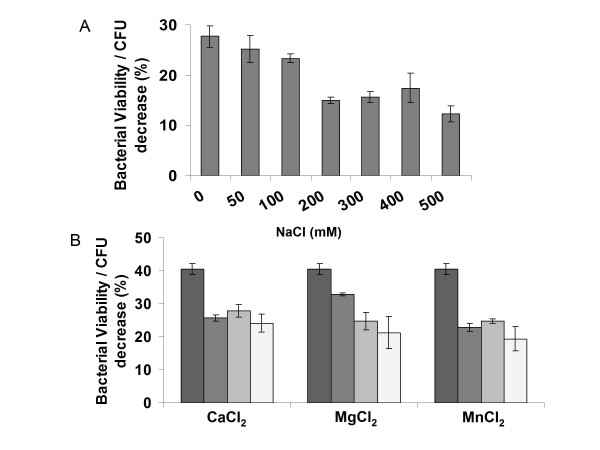
**Effect of NaCl and divalent cations on the antimicrobial activity of HydH5**. A) Activity was determined in 50 mM phosphate buffer containing different NaCl ionic strength. B) Activity was determined in the presence of different concentrations of CaCl_2_, MgCl_2_, and MnCl_2_( 0 mM,  0.75 mM,  1.25 mM,  10.25 mM). Error bars are the means ± standard deviations of three independent assays.

The PG hydrolytic activity of HydH5 was further characterized at several salt concentrations between 50 and 500 mM NaCl, and in the presence of cations (CaCl_2_, MgCl_2 _and MnCl_2_) at concentrations 0.75 to 10.25 mM (Figure 5). The highest activity was obtained at NaCl concentrations lower than 200 mM. All the tested cations inhibited HydH5 activity even at the lowest concentration assayed.

**Figure 6:**
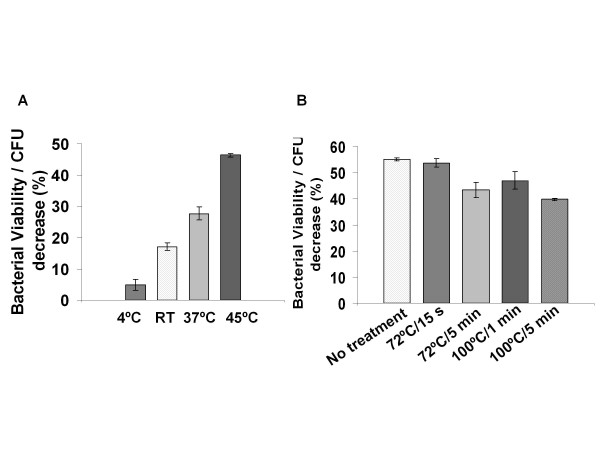
**Influence of temperature on the antimicrobial activity of HydH5**. A) HydH5 (20 μg) activity was tested at room temperature, 4°C, 37°C and 45°C by the standard CFU reduction analysis; B) HydH5 (20 μg) sensitivity to heat treatments (72°C,15 s; 72°C, 5 min; 100°C, 1 min; 100°C, 5 min). After the different treatments, the CFU reduction analysis was performed by challenging *S. aureus *Sa9 cells to the treated HydH5 at 37°C for 30 min. Error bars are the means ± standard deviations of three independent assays.

To assess its thermal stability, HydH5's antimicrobial activity was tested and shown to be maintained at high temperatures (45°C) while lower temperatures decreased its activity (Figure 6A). Aliquots of HydH5 were also heated to 72°C or 100°C followed by cooling to allow refolding and the resultant activity tested at 37°C for 30 min against *S. aureus *Sa9 cells (Figure 6B). HydH5 was not inactivated completely by any of the tested temperature/time combinations. HydH5 activity was detected even after the strongest heat treatment (100°C, 5 min). In this case, a 72% of activity was observed compared to the untreated control.

## Discussion

To combat the emergence of antibiotic resistant pathogens, new strategies are being explored. In this context, the effectiveness of phage-encoded endolysins to eliminate certain infections has been well documented in mouse models [36-38]. The main advantage of these proteins is their ability to kill bacteria with near-species specificity and the reported low incidence of resistance development [36]. Similarly, other phage lytic proteins that also hydrolyze essential PG bonds such as structural PG hydrolases, may also contribute to the supply of new antimicrobials.

Preliminary sequence analyses of the virion-associated PG hydrolase HydH5 revealed two putative lytic domains, namely, N-terminal CHAP domain and LYZ2 domain at the C-terminus. This protein organization resembles that of other phage muralytic enzymes which, similar to endolysins, appear to be modular enzymes containing separate catalytic domains. It has been proposed that the evolution of endolysins, and probably also structural PG hydrolases, has likely occurred through domain swapping and that phage lytic enzymes have co-evolved with host autolysins [39]. In fact, the predicted 3D structure of HydH5 identified another central domain with remote homology to the AmiE catalytic domain of the autolysins AtlE and AtlA, the major *S. epidermidis *and *S. aureus *autolysins, respectively. However, key residue changes seem to have been selected in the active site of HydH5 despite the maintenance of the amidase-like fold, likely rendering a reduced activity amidase domain [28]. Whether or not these mutations have catalytically inactivated the AmiE domain remains to be determined.

It should be noted that LYZ2 domains have been rarely studied in phages, being the phage phiMR11 the only example reported so far [7]. However, it has been predicted that this lysozyme subfamily 2 catalytic domain (SMART accession number: SM00047) is widely distributed in *Staphylococcus *phage, *Staphylococcus *bacteria and other related bacteria.

In this work, we have demonstrated the staphylolytic activity of full-length HydH5 and each of its two catalytic domains by both zymogram analysis and CFU reduction analysis. Having two active catalytic domains decreases the likelihood of resistance development to this antimicrobial in that the pathogen would potentially need two simultaneous mutations in the same cell to become resistant. This is a very attractive trait for potential antimicrobials. Further biochemical analyses are required to definitively assign the endopeptidase and lysozyme activities to these domains and confirm to what extent both contribute to the lytic activity identified in our assays. It has been previously shown that some individual endolysin catalytic domains can lyse *S. aureus *cells in the absence of the complete protein. For example, phi11 and LysK endolysins have active CHAP domain constructs without either the amidase or SH3b domains required [19,30,32].

Our results also indicated that the lytic activity of HydH5 and the two catalytic domains does not require a CBD. Bioinformatic analysis of HydH5 failed to detect a known CBD. It has been speculated that some endolysins possess catalytic domains operating as cell wall-binding domains that direct the protein to target epitopes on the surface of susceptible bacteria [17,40]. There are also numerous reports of C-terminally deleted lysins where the N-terminal lytic domain maintains their staphylococcal- [32] or streptococcal-specificity [41,42] in the absence of their CBD. More surprising are recent studies showing that the lytic activity of the B30 (11) and PlyGBS [43] lysins were maintained or even enhanced, approximately 25-fold, respectively, in engineered lysins in which the SH3 domain has been removed. However, it is not entirely clear which part of the protein determines the specificity. Based on the results that showed binding of the catalytic domains to cells, we hypothesized that substrate recognition in HydH5 might be somehow mediated by its catalytic domains. However, further analyses are required to demonstrate the specificity of this binding for *S. aureus *cells. In this regard, preliminary results about the HydH5 lytic spectrum indicated that most of tested staphylococcal strains were susceptible to this protein (our unpublished results). It should be kept in mind that, in contrast to endolysins, phage structural PG hydrolases might not require a CBD because they are delivered to the PG substrate by the virion particle structure [3].

The proposed function of phage structural PG hydrolases during the first steps of the phage life cycle also implies that their hydrolytic activity should only damage the cell wall slightly in order to avoid premature lysis of the host cell. For this reason, it is not surprising that the lytic activity of HydH5 and both truncated versions were not detected in turbidity reduction assays but were capable of killing *S*. *aureus *Sa9 cells in the CFU reduction analysis. The variable quantitative behaviour of PG hydrolases activities in different lytic assays has also been observed by other authors [44,45]. The killing activity of HydH5 was inhibited by some cations and sodium chloride. Although most of the endolysins described so far has not been tested for the effect of cations, there are some which lytic activity is dependent on or enhanced in the presence of calcium in the assay buffer [32,35,46].

The highest protein activity was detected against actively dividing log phase growth staphylococcal cells, possibly due to a different conformation of the PG. In fact, the degree of peptidoglycan cross-linking is significantly increased in stationary phase cells of species such as *E. coli *and *Bacillus *spp. [47,48]. (An according result) A similar result was observed with the bacteriophage T7 gp16 structural transglycosylase which facilitated infection of *E. coli *cells growing to high cell densities or low temperatures. In these conditions the murein is more highly cross-linked and the internalization of the phage genome is significantly delayed in absence of gp16 protein [6]. Different susceptibility as a function of growth stage was also observed in the Ply700 endolysin [46], which is more active against early and mid-exponential *Streptococcus uberis *cells.

Another feature that is characteristic of HydH5 and other phage structural hydrolases is their thermostability, most likely related to a high refolding capability. HydH5 retained 72% of its activity after a 5-min treatment at 100°C. Likewise, the structural lysozyme from phage phiKMV infecting *Ps. aeruginosa *is also a highly thermostable protein, retaining 26% of its activity after 2 h at 100°C and 21% after autoclaving [47]. By contrast, the lytic activity of most phage endolysins is destroyed by heat treatment [35,41]. This makes structural PG hydrolases attractive antimicrobials to be used in combination with other hygienic procedures based on high temperature such as those applied in food preservation and as structural models for highly thermostable enzymes.

## Conclusions

The lytic activity of HydH5, the virion-associated PG hydrolase from phage phiIPLA88, is due to the presence of two active catalytic domains, namely, an N-terminal CHAP domain and a C-terminal LYZ2 domain. HydH5 lysed *S. aureus *cells in the absence of divalent cations and this activity was optimal against early exponential cells and at 45°C. These characteristics along with its thermostability provide it a potential to be applied as antimicrobial against *S. aureus*.

## Methods

### Bacteria, phages and growth conditions

*S. aureus *Sa9 was isolated from mastitic milk and routinely cultivated in 2 × YT broth at 37°C [22]. *E. coli *DH10B (Gibco, BRL), *E. coli *BL21 (DE3)/pLysS [50] and *E. coli *Rosetta DE3 (Novagen, Madison, USA) were cultivated in 2 × YT broth at 37°C. *E. coli *transformants were selected with 100 μg/ml ampicillin and/or 25 μg/ml chloramphenicol. Bacteriophage vB_SauS-phiIPLA88 (phiIPLA88) was routinely propagated on *S. aureus *Sa9 [22].

### DNA manipulations and plasmids construction

Plasmid DNA was obtained with the High Pure Plasmid Isolation Kit (Roche Diagnostics GmbH, Mannheim, Germany). Analytical and preparative gel electrophoresis of plasmid DNA and restriction fragments was carried out in 0.8% (w/v) agarose-Tris-Acetate horizontal slab gels. Phage phiIPLA88 DNA was extracted and purified as described previously [51]. PCR amplifications were carried out using the PureTaq™ Ready-To-Go™ PCR Beads kit (GE Healthcare, England, United Kingdom) and the PCR fragments were purified using the GenElute PCR clean-up kit (Sigma Missouri, USA). The full-length N-terminally 6×His-tagged protein HydH5 (671 amino acids) was obtained as follows. The primers H1F (5'- GATTGAAATG**GGATCC**ATACATGGG -3') and H2R (5'- CACACCTCT**GAATTC**ATATTTATCTCTTG -3') were annealed to template phiIPLA88 DNA. The resulting PCR product (1934-bp) was cleaved with *BamH*I/*EcoR*I restriction enzymes (sites are bolded in primer sequences; Takara, Otsu, Shiga, Japan) and cloned into plasmid pET-Duet1 (Novagen Madison, USA; pET-Duet1-*orf58*). Truncated HydH5 N-terminally 6×His-tagged derivative containing a 161 amino acid LYZ2 domain was obtained by amplification of *orf58 *with oligonucleotides LYZF (5'- CG**GGATCC**CAAGATACTTAAAGGCAAGGGGA- 3') and LYZR (5'- CACACCTCT**GAATTC**ATATTAATCTCTTG- 3') which generated a 474 bp PCR fragment flanked by the restriction sites *BamH*I and *EcoR*I (as a consequence of the cloning process, 12 additional amino acid residues, Met-Gly-Ser-Ser-His-His-His-His-His-His-Ser-Gln, were introduced at the N-terminal region and 2 amino acid residues, Arg-Asp, at the C-terminal region of the formal 147 amino acid LYZ2 domain defined by the PFAM conserved domain database). Likewise, N-terminal 6×His-tagged CHAP domain was also obtained with the oligonucleotide pair CHAPF (5'- CG**GGATCC**CGAAGTAGTAGAGTGGGC- 3') and CHAPR (5'- G**GAATTC**TTATCTAACAAAATGTGTTACTC -3') yielding a 424 bp PCR product (12 additional amino acid residues, Met-Gly-Ser-Ser-His-His-His-His-His-His-Ser-Gln, were introduced at the N-terminal region of the formal 135 amino acid CHAP domain). Restricted PCR fragments were cloned into plasmid pETDuet-1 (pETDuet1-LYZ2 and pETDuet1-CHAP), respectively. All DNA cloning steps were initially performed in *E. coli *DH10B and then electroporated into *E. coli *BL21(DE3)/pLysS and/or *E. coli *Rosetta (DE3). The integrity of all the clones was verified by both restriction enzyme site profiling and DNA sequence analysis.

### Heterologous overexpression and protein purification

High-level expression of the His-tagged protein HydH5 was achieved in *E. coli *Rosetta (DE3), while the LYZ2 and CHAP HydH5-derived truncations were expressed in *E. coli *BL21(DE3)/pLysS. Exponentially growing cultures (A_600 _0.5) were induced with 1 mM IPTG (isopropyl- beta-D-thiogalactopyranoside). After incubation for 30 min at 37°C, rifampicin was added to a final concentration of 240 μg/ml and incubation continued for 4 h. Cells were pelleted, washed with 50 mM phosphate buffer, pH 7, and frozen at -80 °C. The recombinant proteins were not found in the soluble fraction, and were thus purified from inclusion bodies. Cell pellets from 1.2 l cultures were resuspended in 10 ml per g of wet weight of 1× cell resuspension buffer (iFOLD Protein Refolding System 2) (Novagen, Madison, USA) and sonicated on ice (15×5 s pulses with 15 s recovery between pulses) following the manufacturer's instructions. Inclusion bodies containing HydH5, LYZ2 and CHAP proteins were obtained via centrifugation (8000 × *g*) and stored as pellets at -80°C. They were denatured in iFold Guanidine denaturation buffer and optimal conditions for correct folding (highest activity and solubility) were determined with the iFold protein refolding matrix and via antimicrobial assays. The highest activity and solubility was obtained by refolding HydH5 and LYZ2 in buffer A (HEPES 50 mM, NDSB-201 0.5 M, CaCl_2 _0.25 mM, MnCl_2 _0.25 mM, MgCl_2 _0.25 mM, TCEP 1 mM, NaCl 24 mM, KCl 1 mM pH 7.5) and CHAP in buffer B (TAPS 50 mM, NDSB-256 0.5 M, NaCl 24 mM, KCl 1 mM pH 8.5). Fractions containing HydH5, LYZ2 and CHAP proteins were diluted in glycerol (50% final concentration), and stored at -80°C. Purity of each preparation was determined in 15% (w/v) SDS-PAGE gels. Electrophoresis was conducted in Tris-Glycine buffer at 30 mA for 1 h in a BioRad Mini-Protean gel apparatus (BioRad, Hercules, CA). Protein was quantified by the Quick Start Bradford Protein Assay (BioRad, Hercules, CA).

### Determination of the lytic activity

Antimicrobial activity was determined by the CFU reduction analysis against *S. aureus *Sa9 strain. Exponentially growing cells (A_600 _0.5) were recovered by centrifugation, washed and resuspended in 50 mM phosphate buffer, pH 7 to A_600 _0.1. Then, 20 μg of protein (HydH5, CHAP or LYZ2) were mixed with 4×10^6 ^CFU/ml and incubated at 37°C for 30 min. All these experiments were performed in triplicate. Serial dilutions were plated in triplicate on Baird-Parker agar plates, and survival was determined after 18 h at 37°C. Buffer alone controls were included in the analysis. The antimicrobial activity was expressed as the bacterial viable counts decrease. This value was calculated as the dead percentage referred to an untreated control. Likewise, the ability of HydH5 to kill *S. aureus *Sa9 cells at different stages of growth, its stability under different thermal treatments and the influence of NaCl and different cations were also tested using this assay. *S. aureus *Sa9 cells were harvested at different times throughout growth: early (A_600 _0.2), mid-exponential (A_600 _0.55), late exponential (A_600 _2), and stationary (A_600 _3), washed and resuspended in 50 mM phosphate buffer, pH 7 to A_600 _0.1, and treated as described above. The influence of temperature on enzyme activity was tested by challenging *S. aureus *Sa9 cells with HydH5 enzyme at different temperatures (4°C, 20°C, 37°C, 45°C) for 30 min and compared to control samples without protein incubated in the same conditions. Temperature stability was tested by incubating HydH5 (20 μg) at variable temperatures and times (72°C 15 s, 72°C 5 min, 100°C 1 min, 100°C 5 min) previously to the *S. aureus *Sa9 cells challenging.

### Zymogram analysis

To detect HydH5, CHAP and LYZ2 domains activities, zymogram assays were performed using identical 10 ml 15% (w/v) SDS-PAGE with or without *S. aureus *Sa9 cells from a 300 ml culture (A_600 _0.5) embedded in the zymogram. Samples were prepared according to standard SDS-PAGE sample preparation [52]. Gels were run at 30 mA for 1 h in a Bio-Rad Mini-Protean gel apparatus. SDS gels were stained via conventional Coomassie staining. Zymograms were soaked for 30 min in distilled water to remove SDS and then overnight incubated at room temperature in distilled water to detect areas of clearing in the turbid gel.

### Cell wall binding assay

*S. aureus *Sa9 was grown to an exponential phase (A_600 _0.8), harvested by centrifugation, concentrated 10-fold in PBS (100 mM NaCl, 3 mM KCl, 10 mM NaH_2_PO_4_, 2 mM KH_2_PO_4_, pH 7.5), and stored on ice. 5 μg of protein was added to 100 μl of cell suspension and incubated at 37°C for 10 min. The suspension was centrifuged at 10,000 × *g*, the pellet washed three times with PBS buffer, resuspended in 50 μl of PBS, boiled with the sample buffer [52] and analysed with 15% (w/v) SDS-PAGE. Electrophoresis was conducted in Tris-Glycine buffer at 30 mA for 1 h. Unbound (present in the supernatant) and cell- bound protein were assayed by western-blotting using monoclonal antibodies against His-tag (Sigma, Missouri, USA) following the manufacturer's instructions.

### Bioinformatic analysis

Phylogenetic position of the full length HydH5 protein was determined by the Neighbor-Joining method. The evolutionary distances were computed using the Poisson-correction method and expressed in the units of the number of amino acid substitutions per site. All positions containing gaps and missing data were eliminated from the dataset. Phylogenetic analyses were conducted in MEGA4 [53]. To predict the three-dimensional (3D) structure of HydH5, remote homology templates were identified by a search of HydH5 sequence against PDB database implementing in HHpred server [54]. Template-based protein structure modelling was done according to MODELLER [55].

## Authors' contributions

All authors reviewed and approved the final version of the manuscript. LR and PG conducted the protein analysis. YZ performed bioinformatics analyses. DMD supervised the work in USA. PG, BM and AR designed the study, obtained funding and wrote the manuscript.

## Competing interests

The authors declare that they have no competing interests.
